# Regulation of SPDEF expression by DNA methylation in advanced prostate cancer

**DOI:** 10.3389/fendo.2023.1156120

**Published:** 2023-10-11

**Authors:** Mousa Vatanmakanian, Joshua J. Steffan, Sweaty Koul, Augusto C. Ochoa, Lakshmi S. Chaturvedi, Hari K. Koul

**Affiliations:** ^1^Department of Biochemistry & Molecular Biology, School of Medicine, Louisiana State University Health Sciences Center, New Orleans, LA, United States; ^2^LSU-LCMC (Louisiana Children's Medical Center) Cancer Center, School of Medicine, Louisiana State University Health Sciences Center, New Orleans, LA, United States; ^3^Program in Urosciences, Division of Urology, Department of Surgery, School of Medicine, University of Colorado Anschutz Medical Campus, Aurora, CO, United States; ^4^Department of Interdisciplinary Oncology, School of Medicine, Louisiana State University Health Sciences Center, New Orleans, LA, United States; ^5^Department of Urology, School of Medicine, Louisiana State University Health Sciences Center, New Orleans, LA, United States; ^6^Southeast Louisiana Veterans Health Care System, New Orleans, LA, United States

**Keywords:** SPDEF, prostate cancer, CRPC, DNMT, epigenetics, DNA methylation, plasticity

## Abstract

**Introduction:**

Prostate cancer (PCa) presents a significant health challenge in men, with a substantial number of deaths attributed to metastatic castration resistant PCa (mCRPC). Moreover, African American men experience disproportionately high mortality rates due to PCa. This study delves into the pivotal role of SPDEF, a prostate specific Ets transcription factor, and its regulation by DNA methylation in the context of PCa progression.

**Methods:**

We performed Epigenetic reprogramming using daily treatment with non-toxic dose of 5Aza-2-deoxycytidine (5Aza-dC) for two weeks to assess its impact on PDEF expression in prostate cancer cells. Next, we conducted functional studies on reprogrammed cells, including cell migration (wound-healing assay), invasion (Boyden-Chamber test), and proliferation (MTT assay) to comprehensively evaluate the consequences of altered PDEF expression. We used bisulfite sequencing (BSP) to examine DNA methylation at SPDEF promoter. Simultaneously, we utilized siRNA-mediated targeting of key DNMTs (DNMT1, DNMT3A, and DNMT3B) to elucidate their specific role in regulating PDEF. We measured mRNA and protein expressions using qRT-PCR and immune-blotting techniques, respectively.

**Results:**

In this report, we observed that: a) there is a gradual decrease in SPDEF expression with a concomitant increase in methylated CpG sites within the SPDEF gene during prostate cancer progression from lower to higher Gleason grade; b) Expression of DNMT’s (DNMT1, 3a and 3b) is increased during prostate cancer progression, and there is an inverse correlation between SPDEF and DNMT expression; c) SPDEF levels are decreased in RC77/T, a line of PCa cells from African American origin similar to PC3 and DU145 cells (CRPC cells), as compared to LNCaP cells , a line of androgen dependent cells,; d) the 5′ CpG island of SPDEF gene are hypermethylated in SPDEF-negative CRPC ( PC3, DU145 and RC77/T) cell lines but the same regions are hypomethylated in SPDEF-positive castrate sensitive (LNCaP) cell line ; (e) expression of SPDEF in PCa cells lacking SPDEF decreases cell migration and invasion, but has no significant effect on cell proliferation, and; (f) treatment with the demethylating agent, 5-aza-2′-deoxycytidine, or silencing of the DNMT’s by siRNA, partially restores SPDEF expression in SPDEF-negative PCa cell lines, and decreases cell migration and invasion.

**Discussion:**

These results indicate hypermethylation is a prevalent mechanism for decreasing SPDEF expression during prostate cancer progression. The data demonstrate that loss of SPDEF expression in prostate cancer cells, a critical step in cellular plasticity, results from a potentially reversible process of aberrant DNA methylation. These studies suggest DMNT activity as a potential therapeutic vulnerability that can be exploited for limiting cellular plasticity, tumor progression, and therapy resistance in prostate cancer.

## Graphical Abstract

Prostate cancer progression is associated with increased expression of DNMTs and a consequent increased methylation on SPDEF gene and subsequent decrease in SPDEF expression. Inhibition of DNMTs restores SPDEF expression and results in phenotype reversal.

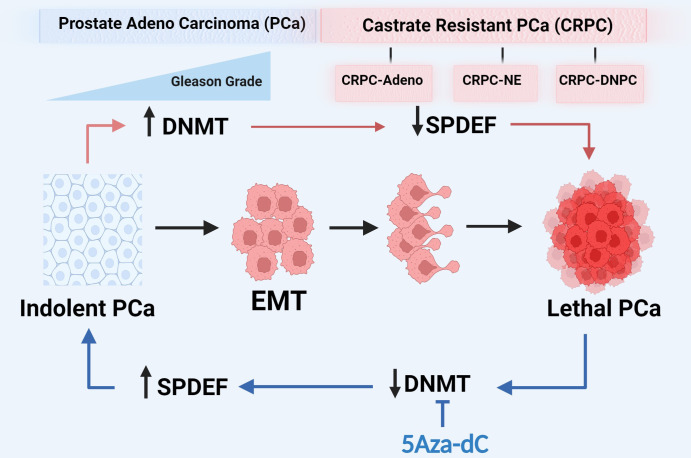

## 1 Introduction

Prostate cancer (PCa) is the most common non-cutaneous cancer diagnosed and the second leading cause of cancer-related deaths among men in the USA (1, 2). Moreover, African American men have an earlier onset, higher aggressiveness, more extensive metastases, and increased mortality rates compared to Caucasian (CA)men (3). Conventional therapies produce a high rate of cure for patients with localized prostate cancer and ten-year survival for the localized disease of 98%. However, once patients develop metastasis, androgen deprivation therapy (ADT) is the principal treatment. Moreover, despite an initial response to ADT, many patients develop castration-resistant phenotype (mCRPC) (4–8). Although currently approved treatments of mCRPC with androgen receptor signaling inhibitors (ARSI) are effective initially, they do not provide durable responses (9–13). At present, there is no cure for the patients who develop mCRPC.

Progression of PCa to mCRPC and development of therapeutic resistance requires the deregulation of AR signaling and the gain of cell motility and invasiveness. Cellular plasticity has been identified as an essential mechanism of treatment resistance in prostate cancer (14),. Cellular plasticity can drive epithelial–mesenchymal transition (EMT), a reversible cellular process contributing to tumor progression, invasion, metastasis, and resistance to therapy (15). These changes include enhanced motility and invasion. Members of the ETS (E-Twenty-Six) family of transcription factors play an essential role in regulating many biological processes, including cellular differentiation and plasticity (16). Prostate-Derived Ets Factor/Sam Pointed Domain Ets Factor (SPDEF/SPDEF) is an Ets protein family transcription factor. Perturbation of ETS factor activity leads to tumor initiation, progression, and metastasis (17). SPDEF is expressed in the epithelial layer of the lumen containing organs, including the prostate, breast, and colon (18–20). SPDEF has been characterized as a cell identity-related super-enhancer-driven transcription factor in the luminal PCa cell line, LNCaP (21). Prior studies reported that SPDEF expression decreases during prostate and breast tumor progression (22, 23). Loss of SPDEF is associated with PCa progression and increased cell migration and invasion *in vitro* and metastasis *in vivo* (24–26). Our recent studies suggest that SPDEF plays a crucial role in PCa progression and metastasis in part by restricting cellular plasticity and promoting luminal epithelial phenotype (27). However, the expression of SPDEF in prostate cancer cells of African-American Origin has not been investigated. Moreover, the mechanisms by which SPDEF expression decreases during prostate cancer progression are not delineated.

Epigenetic mechanisms such as DNA methylation and histone modifications have long been associated with prostate cancer (28, 29). The most common epigenetic program, DNA methylation at CpG islands, is one of the regulatory mechanisms of gene expression in prostate cancer (30). DNA methylation leads to gene silencing by limiting access of transcription factors to target binding sites (31) or by recruitment of methyl-binding domain proteins, which facilitate the assembly of chromatin condensation into transcriptionally repressive conformations (32, 33). DNA methylation is catalyzed by DNA methyltransferases (DNMTs), including DNMT1, DNMT3a, and DNMT3b, and hypermethylation at CpG islands leads to the loss of gene expression (34–36). However, the role of DNMTs in the regulation of SPDEF has yet to be investigated.

The work herein is a follow-up to our previously published studies of the relationship between SPDEF expression and prostate carcinogenesis (27). We observed that similar to PC3 and DU145 cells (CRPC cells), SPDEF levels are decreased in RC77/T, a line of PCa cells of African American origin, compared to LNCaP cells, a line of androgen-dependent cells. In addition, our analysis of gene expression in prostate cancer specimens in human prostate cancer datasets revealed that SPDEF mRNA expression is decreased during prostate cancer progression. Moreover, we observed that high Gleason-grade prostate tumors have higher levels of DNMTs (DNMT1, DNMT3A, and DNMT3B) and frequently contain highly methylated CpG sites within the *SPDEF gene.* In addition, our findings indicate that the CpG islands of the SPDEF gene are hypermethylated in CRPC cell lines.

In contrast, the CpG islands of the SPDEF gene are hypomethylated in LNCaP cells. SPDEF expression in CRPC (PC3 and DU145) cells, castrate sensitive cells (LNCaP) cells, and RC77/T, African American PCa cells correlated with the methylation patterns at CpG islands of the SPDEF gene. These results suggest that DNA methylation may be pivotal in decreasing the SPDEF gene expression in PCa cells. Our finding further confirms the conclusion that culturing PCa cells that exhibit hypermethylation of CpG islands of the SPDEF gene for several cycles in sub-lethal levels of 5-Aza-dC (DNMT inhibitor) partially restores SPDEF mRNA and protein expression. Furthermore, such treatment regiments with 5-Aza-dC also inhibited cell migration and invasion similar to that observed with SPDEF expression. Our results suggest that epigenetic reprogramming with DNMT inhibitors could restore SPDEF expression and thereby limit cellular plasticity in PCa. Preliminary reports have been presented and published in abstract format (37–39).

## 2 Materials and methods

### 2.1 Cell culture and stable cell line establishment

DU145, RC77/T (and its derivatives), and phoenix cells were maintained in Dulbecco’s Modified Eagle Medium (DMEM, Gibco #10569-010), PC3 (and its derivatives) cells were maintained in DMEM/F12 (Corning # 23-10-090-CM), LNCaP cells were cultured in RPMI-1640 (Corning #10-040-CV). All media were supplemented with 10% heat-inactivated fetal bovine serum (FBS), Cat# 10437028- Gibco; 1% penicillin-streptomycin, Cat# 15070-063-Gibco; and were maintained at 37°C, 5% CO2 in a humidified incubator. To mimic androgen deprivation therapy on LNCaP cells, the phenol red-free RPMI-1640 (Gibco#11835030) medium was supplemented with 10% charcoal-stripped heat-inactivated FBS (CS-FBS) (biowest #S162C) and 1% penicillin-streptomycin.

### 2.2 Generation of SPDEF overexpression RC77/T cells

RC77/T-SPDEF and RC77/T-VC cells were generated by retroviral transfection protocol similar to PC3-SPDEF, and PC3-VC described earlier (24, 27, 40). Briefly, SPDEF was cloned, linked with an amino-terminal FLAG tag, and inserted into the pBABE retroviral vector. Phoenix cells stably expressing retroviral packaging proteins were transfected with pBabe-VC and pBabe-SPDEF using Lipofectamine 3000 transfection kit (Invitrogen# L3000-008) per the manufacturer’s protocol. The supernatants were collected and filtered by a 0.45µm pore size filter, mixed with 16µM polybrene, and used to transduce PC3 and RC77/T cells for three rounds every 12 hours. Puromycin selection was initiated and continued for three weeks to generate stable cell lines. Mycoplasma PCR test (Abcam cat#G238) was performed routinely to ensure contamination-free cell line generation.

### 2.3 RNA extraction and quantitative real-time PCR (qRT-PCR)

Total RNA was extracted from the cells using RNesay® Mini Kit (QIAGEN #74106) per the manufacturer’s recommendation. The concentration and purity of RNA were confirmed by Nano-drop spectrophotometer. Complementary DNA (cDNA) was generated using an iScript cDNA synthesis kit (Bio-Rad #1708891). The iTaqTM Universal SYBER® Green Supermix (Bio-Rad #172-5121) was used to amplify the transcripts. The quantitative real-time PCR (qRT-PCR) was performed using the LightCycler480 (Roche) as described previously (41). The qRT-PCR results were analyzed with the delta-delta Ct (2^-ΔΔ*CT*^) method normalized with the housekeeping β-Actin gene (internal control). The primers used in this study are listed in the **Supplemental Table S1**.

### 2.4 Protein extraction and western blotting

Protein extraction and western blotting were performed as described previously with slight modifications (42). Briefly, cells were directly lysed with RIPA buffer (150mM NaCl, 10mM Tris-HCl pH 7.5, 0.1% SDS, 1% Triton X-100, 5mM EDTA pH 8.0, 1% sodium deoxycholate, and 4mM sodium fluoride) containing 1mM sodium orthovanadate, 1mM dithiothreitol (DTT), 1mM cocktail of protease inhibitors (Sigma # 539133), and 1mM phenyl-methyl-sulfonyl fluoride (PMSF). Protease inhibitors were freshly added to the RIPA. After 20 minutes of incubation on ice, the lysates were sonicated and centrifuged at 14,000 rpm for 15 minutes at 4°C. The supernatant was transferred to a new tube, and the protein was quantified using the bicinchoninic acid assay (BCA) method (Thermo #23227). Forty micrograms of each protein sample were electrophoresed on a gradient polyacrylamide gel (4-15% sodium dodecyl sulfate-polyacrylamide gel electrophoresis (SDS-PAGE, Bio-Rad, #456-1093). Proteins were transferred to 0.45µm Polyvinylidene fluoride (PVDF) membrane, and non-fat dry milk was used to block the non-specific binding, followed by incubation with primary and HRP-coupled secondary antibodies to identify the proteins of interest. The chemiluminescent ECL solution (# Immobilon® Forte #WBLUF0500 Millipore Sigma) was used to visualize the blots via the VersaDoc imaging system (Bio-Rad). The primary antibodies used were: anti-SPDEF (Santa Cruz Biotechnology sc-166846), anti-EpCAM (Cell Signaling #93790), anti-zeb1 (Santa Cruz #sc-515797), anti-vimentin (Cell Signaling #5741S), anti-E-cadherin (Cell Signaling #3195), anti-N-cadherin (Cell Signaling #13116S), anti-twist1 (Cell Signaling #46702), anti-claudin (Cell Signaling #4933), anti-snail (Cell Signaling #3879), anti-beta-catenin (Cell Signaling #9581S), anti-DNMT1 (Cell signaling #50332), anti-DNMT3A (Cell signaling #3598), anti-DNMT3B (Cell signaling #57868), anti-P21 (Cell signaling #2946), anti-GAPDH (monoclonal mouse; Sigma #G8795), and anti-α-Tubulin (Santa Cruz #sc5286) and anti-B-Tubulin (Santa Cruz #sc166729). HRP-linked anti-mouse IgG and anti-rabbit IgG secondary antibodies were obtained from Jackson Lab (#115-035-003 and # 111-035-003, respectively).

### 2.5 DNA extraction and bisulfite conversion

The genomic DNA was isolated from the cell cultures using the Wizard® Genomic DNA purification kit (Promega #A1125) per the manufacturer’s recommendations. DNA was quantified using a Nano-drop spectrophotometer and ensured to have enough concentration and purity optimal for efficient bisulfite conversion (the concentration of more than 200 ng/μl, OD260/230 of 2.0–2.2, and 260/280 of 1.8–2.0). A 200-500ng of starting DNA was used in the bisulfite modification reaction using EpiJET Bisulfite Conversion Kit (Thermo scientific #K1461). The sodium bisulfite catalyzes a reaction in which all the unmethylated cytosine (uC) nucleotides convert to uracil (U) or thymidine (T) and leaves the methylated cytosine (mC) intact.

### 2.6 Primer design and bisulfite sequencing PCR (BSP)

The MethPrimer 2 online tool (https://www.urogene.org/methprimer2/) was used to predict the CpG island based on the promoter sequence provided by the UCSC database, and Bisulfite Specific PCR (BSP) primers were designed to cover 200-400bp and synthesized by Integrated DNA Technologies, Inc. (IDT). Primers used are listed in the **Supplemental Table S1**. The PCR reactions were performed using TaKaRa EpiTaq HS (for bisulfite-treated DNA, #R110B). The Taq polymerase provided in the kit remains a hanging Adenosine (A) on both flanks of the amplicon. Briefly, the PCR mixture containing 5μl of 10X buffer, 2.5μM MgCl2, 10μM dNTP, DNA (approximately 50ng), 2μl of each forward and reverse primers (10μM each), and 0.5μl of Taq polymerase, were diluted with water to the final volume of 50μl. The reactions were conducted in a T100 Thermal Cycler device (BIO-RAD) with a primary denaturation for 5 min at 95°C followed by 40 cycles of denaturation at 94°C for 30 Sec, 55°C for 30 Sec, 72°C for 40 Sec, and a final extension at 72°C for 10 min. The PCR products were electrophoresed on 1.5% agarose gel to ensure successful amplification, and then it was purified using the QIAquick PCR purification kit (QIAGEN, #28106). Using the TA cloning method, thirty nanograms of the resulting products were cloned into the pGEM®-T Easy Vector System (Promega, # A1360). The ligation reaction was incubated overnight at 4°C and then transformed to TOP-10 competent E. coli (Invitrogen #c404003). The bacteria were grown overnight on LB agar plates supplemented by 100µg/ml ampicillin 0.5mM IPTG and 80µg/ml X-Gal, giving rise to blue/white colonies. 5-10 positive colonies (whites) were selected from each plate and amplified overnight on LB medium. The plasmids were extracted via QIAprep spin miniprep kit (QIAGEN, #27106) and were submitted for Sanger sequencing to LSU-LCMC cancer center genomics core to be sequenced using M-13 reverse primer. The obtained sequences were analyzed via Biq-Analyzer software and QUMA, a Quantification tool for methylation analysis (https://wwwquma.cdb.riken.jp/).

### 2.7 MTT cell proliferation and cytotoxicity assay

To monitor the cell proliferation as well as response to the treatments, we used either the MTT reagent (Millipore Sigma # 57360-69-7) or Cell Counting Kit-8 (CCK-8) kit (cat# NC9261855, Dojindo Molecular Technologies, Inc). Briefly, cells were seeded on 96-well plates at 3000 cells/well density in 100µl of their growth medium and treated as indicated. Twenty microliters of the MTT reagent (5mg/ml) were added to the cells and incubated in a CO2 incubator for 4 hours. The entire components of the wells were gently aspirated, and the dye crystals were dissolved in 50-100µl DMSO, resulting in a purple color, the density of which was measured by SYNERGYMx Microplate Reader at 590nm wavelength. We used the CCK-8 kit in some experiments per the manufacturer’s recommendations.

### 2.8 Invasion assay

Invasion assay was performed as previously described (43), and the results were quantified using Image J software. The quantified data were presented as cell count.

### 2.9 Wound healing assay

A wound healing assay was performed by seeding cells in 6-well plates at a high density and allowing them to form cell monolayers overnight, as described previously (27). A 200μL sterile plastic tip was used to create a wound line across the surface of the plate, and the cells were washed with PBS. Cell migration was recorded at 0, 12, 24, 48, and 72 hours and quantified by Image J. The quantified data are presented as the mean of area subtracted from time 0 for each time point.

### 2.10 Colony formation assay

The colony formation assay was performed by seeding 300 cells per well in a 6-well plate grown for seven days. The cells were stained with crystal violet. The data were quantified by Image J software and presented as colony count, the average size of the colonies, and percent (%) area.

### 2.11 siRNA (short interfering RNA) transfection

All the siRNA for DNMT1, DNMT3A, and DNMT3B (DNMT1: # SASI_Hs01_00204021, #SASI_Hs01_00204022; DNMT3A: # SASI_Hs01_00160192, # SASI_Hs01_00160193; DNMT3B: # SASI_Hs01_00016756, # SASI_Hs01_00016757; and Scramble Control: # SIC001 SINRA UNIV NEGATIVE CONTROL #1) were purchased from Millipore SIGMA. For each gene (DNMT1, DNMT3A, and DNMT3B), we used a pool of two siRNAs to suppress the corresponding gene at two different concentrations (25 and 50nM). The transfection was performed using Lipofectamine 3000 transfection kit (Invitrogen# L3000-008) according to the manufacturer’s protocol.

### 2.12 Clinical data analysis

The pcan normalized gene expression, methylation beta values, as well as phonotypes including overall survival time, and Gleason scores were downloaded from the University of California, Santa Cruz (UCSC) genome browser (https://xena.ucsc.edu/), The Cancer Genome Atlas Prostate Adenocarcinoma (TCGA-PRAD). Data for clinical cohorts DKFZ prostate cancer and NEPC datasets were downloaded from cBioPortal (https://www.cbioportal.org/) (44, 45). The heat maps representing the expression and methylation levels are generated within the UCSC genome browser tool.

### 2.13 Statistical analysis

All experiments were conducted in triplicate unless otherwise stated. The *in vitro* assays were repeated in three independent biological replicates to ensure reproducibility. For gene expression data, t-tests and linear regression analysis were used to compare each group separately versus control and to analyze correlations between gene expression and clinical variables using GraphPad Prism software. Normality tests were conducted for all gene expression data and found to be normally distributed, allowing for the use of parametric tests. For colony formation, proliferation, migration, and invasion tests, t-tests were used to compare each group separately with respective controls. One-way ANOVA compared the baseline expression differences of SPDEF, DNMT1, DNMT3A, and DNMT3B in different cell lines. For DNA methylation data, non-parametric statistical tests were used due to the non-normal distribution of methylation status. The Mann-Whitney U-test was used to evaluate the statistical significance between two groups of CpG sites across the SPDEF gene. Fisher’s exact test was used to determine the significance of the difference between two bisulfite sequence groups at each CpG site. Correlations between gene expression, methylation levels, and Gleason score in clinical data were analyzed using linear regression analysis. Unless otherwise stated, data are presented as mean ± Standard error of the mean (MEAN ± SEM) on the graphs. P values of less than 0.05 were considered to be statistically significant. We also conducted appropriate normality tests for all gene expression data. We found them to be normally distributed, allowing for the use of parametric tests such as t-tests and linear regression analysis.

## 3 Results

### 3.1 PCa progression is associated with decreased SPDEF

**Figure 1:**
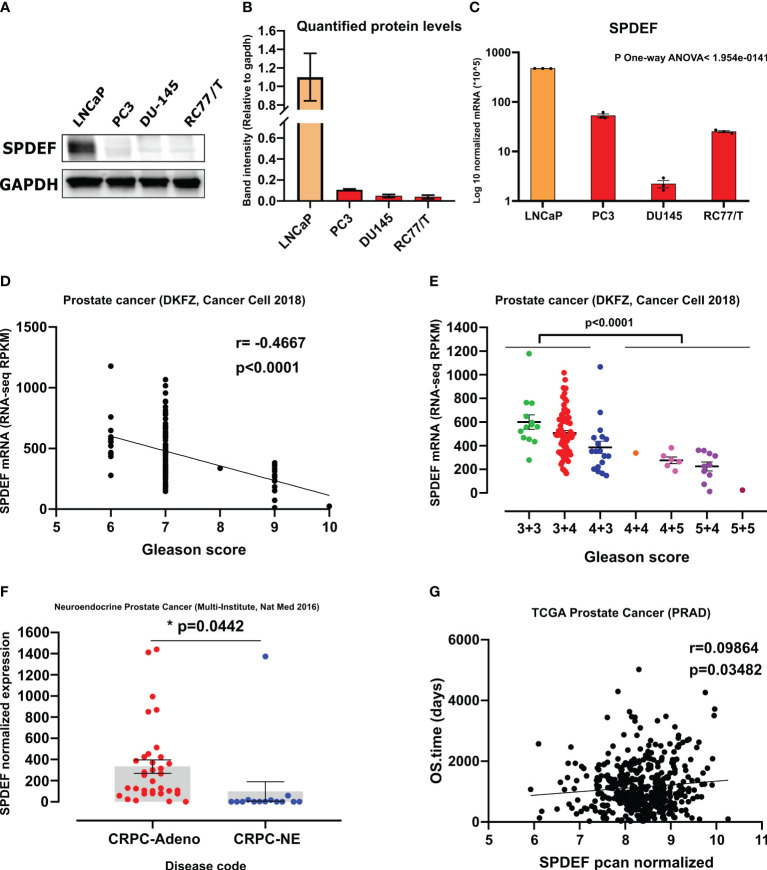
SPDEF expression is decreased during prostate cancer progression: **(A)** Immunoblot showing protein expression of SPDEF in the same cell lines. **(B)** Quantified amounts of protein bands in the immunoblot. **(C)** SPDEF baseline mRNA levels in multiple PCa cell lines measured by qRT-PCR. **(D, E)** data showing the association of SPDEF mRNA levels (RPKM) with Gleason score in clinical cohorts of human Prostate Cancer (DKFZ, Cancer Cell 2018). **(F)** SPDEF mRNA levels in CRPC-Adeno vs. CRPC-NE in from the multi-institutional NEPC data set, and **(G)** The association of SPDEF pcan normalized values with the overall survival time in TCGA Prostate Cancer (PRAD). p<0.05*.

We performed gene expression analysis using qRT-PCR to measure the mRNA levels of the SPDEF and Western blot analysis to measure protein levels of SPDEF in multiple PCa LNCaP, PC3, DU145, and RC77/T cell lines. The results (**Figures 1A, B**) show that SPDEF expression was significantly higher in LNCaP cells than in RC77/T, PC3, and DU145 cells. Similarly, we observed decreased SPDEF mRNA transcripts in RC77/T, PC3, and DU145 cell lines compared to LNCaP (**Figure 1C**). To more thoroughly investigate the association of SPDEF in PCa progression, we opted to analyze human clinical cohorts of prostate cancer. Interestingly, our analysis of prostate cancer (DKFZ, Cancer Cell 2018) showed a significant negative correlation between SPDEF transcripts and Gleason score; patients with higher Gleason scores showed significantly reduced levels of SPDEF (n=119, r=0.4667, p<0.0001, **Figures 1D, E**). As depicted in **Figure 1F**, we also analyzed data from the neuroendocrine prostate cancer clinical cohort (45). Our results show that SPDEF mRNA levels in patients with CRPC neuroendocrine phenotype are reduced dramatically. Moreover, the SPDEF pcan normalized expression was significantly correlated with overall survival time (n=568, r=0.09864, p=0.035, **Figure 1G**) in the TCGA prostate cancer (PRAD) clinical cohort. Our *in-vitro* and clinical cohort analysis of human prostate cancer suggest that decreased expression of SPDEF highly correlates with PCa disease progression.

### 3.2 SPDEF expression is inversely correlated with DNA methyl transferases in prostate cancer

**Figure 2:**
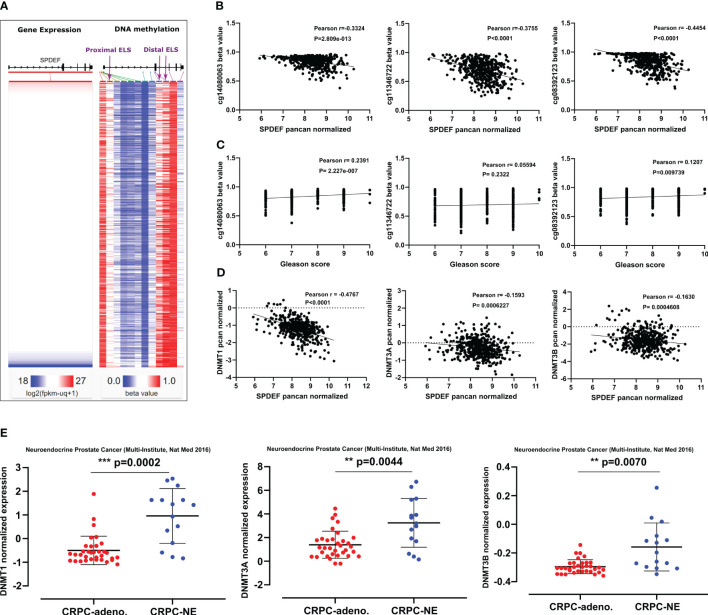
SPDEF expression is inversely related to methylation on CpG islands in the *SPDEF* gene: **(A)** Heat map of SPDEF mRNA expression along with the CpG methylation map of *SPDEF* gene (beta values) in the TCGA prostate cancer (PRAD) showing an inverse correlation at three major CpG- rich regions of our interest (purple arrows, proximal and distal enhancer elements) and SPDEF expression. **(B)** The correlation of SPDEF pcan normalized expression with the three major CpG-rich regions methylation beta values. **(C)** Gleason scores correlate with methylation on three major CpG-rich regions of *SPDEF* gene beta values. **(D)** The correlation of SPDEF pcan normalized expression with DNMT1 (left panel), DNMT3A (middle panel), and DNMT3B (right panel) pcan normalized expression values from TCGA prostate cancer (PRAD) cohort. **(E)** The t-test analysis of normalized expression of DNMT1 (left panel), DNMT3A (middle panel), and DNMT3B (right panel) between CRPC-Adeno versus CRPC-NE phenotype from the multi-institutional NEPC data set. p<0.01**, p<0.001***.

DNA methylation at CpG islands is the well-studied epigenetic mechanism contributing to silencing the genes. Because SPDEF expression is decreased during prostate cancer progression in patients and advanced PCa cell lines, we hypothesized that the SPDEF gene might be partially regulated via DNA hypermethylation. To investigate this, we studied the correlation of SPDEF expression levels with the DNA methyltransferase enzymes in the TCGA Prostate Cancer (PRAD) clinical cohort (n=568). The heat map in **Figure 2A** depicts the SPDEF gene expression and DNA methylation beta values on the cohort patients. Interestingly, the SPDEF expression showed a significant inverse correlation with three CpG-rich regions where the CpG islands are predicted (**Figures 2A, B**), one close to the promoter-like signature (PLS) and proximal enhancer-like element (cg14080063, r=-0.3324, p=2.809e-013) and two in the proximity of multiple distal enhancer elements (cg11346722, r=-0.3755, p<0.0001; and cg08392123, r=0.4454, p<0.0001) (**Figure 2B**). Interestingly, methylation at these sites was found to be directly correlated with Gleason scores in the patients (**Figure 2C**). Next we evaluated the expression of the enzymes transferring the methyl groups to the CpG sites including DNMT1, DNMT3A, and DNMT3B in the patients. Remarkably, our correlation analysis showed a negative correlation between SPDEF normalized expression and all three DNA methyltransferases (**Figure 2D**), suggesting that SPDEF might be at least in part regulated by DNA hypermethylation. Furthermore, analysis of other datasets, including prostate adenocarcinoma (TCGA, Firehose legacy), TCGA prostate cancer (PRAD), and GDC TCGA prostate cancer (n=632), also confirmed these data (**Supplemental Figure S1**). Interestingly, analysis of data in the Multi-Institutional NEPC dataset (45) revealed that CRPC-NE patients’ samples are significantly enriched in DNMT1, DNMT3A, and DNMT3B transcripts compared to CRPC-Adeno patients (**Figure 2E**). Collectively, these results led us to investigate the effects of DNA methylation on regulating SPDEF levels in PCa cell lines with varying levels of SPDEF.

### 3.3 *SPDEF* gene is hypermethylated at CpG islands in CRPC cell lines and the African American PCa cell line compared to LNCaP cells

**Figure 3:**
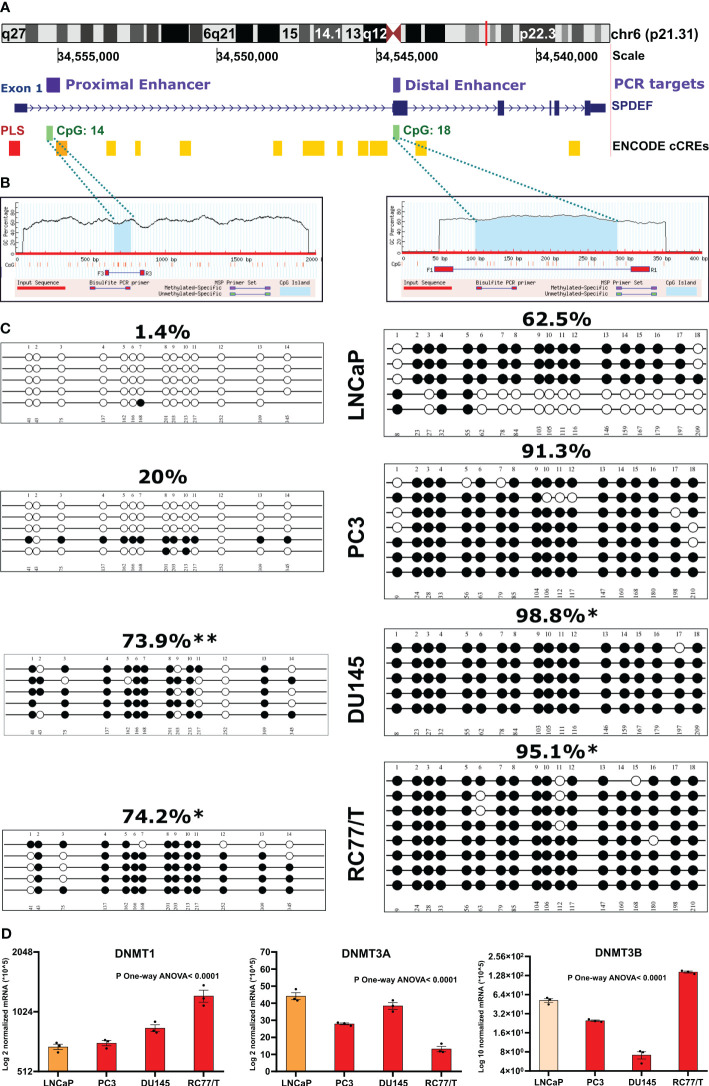
Methylation of *SPDEF* gene in prostate cancer cell lines: **(A)** Schematic representation of *SPDEF* gene (blue) and cis-regulatory elements including promoter-like signature (PLS, red bar) (red), proximal enhance-like signature (ELS, orange bar), and distal enhancer-like signatures (yellow bars), and CpG islands (green). **(B)** CpG island prediction from SPDEF genomic regions, proximal (left) and distal enhancer (right) showing the CpG island as blue shades, CpG sites as red bars, and the location of primers designed for Bisulfite sequencing (BSP) experiment. **(C)** Lollipops representation of methylation data on multiple PCa cell lines performed via BSP experiment showing the percentage of CpG sites’ methylation on proximal (left panels) and distal (right panels) enhancer. **(D)** mRNA expression of DNMT1 (left panel), DNMT3A (middle panel), and DNMT3B (right panel) in multiple PCa cell lines. P Mann Whitney U- test p<0.05*, p<0.01**.

To confirm the role of DNA methylation in regulating SPDEF levels in PCa progression, we opted to measure the methylation levels of *SPDEF* CpG islands in PCa cell lines with known SPDEF expression levels. We utilized the UCSC genome browser to predict the CpG islands and spot the candidate cis-regulatory elements (cCREs) within the SPDEF gene. These elements have been annotated through the GENECODE project. cCREs with promoter-like signatures (cCRE-PLS) typically fall within 200 bp of an annotated GENCODE transcriptional start site (TSS). Interestingly, the browser predicted a 336bp promoter-like signature (PLS) starting upstream of the SPDEF gene, spanning the exon 1 (-188 to +146) (**Figure 3A**, the red bar). Moreover, the gene was associated with numerous proximal (**Figure 3A**, orange bars) and distal enhancer-like elements (**Figure 3A**, yellow bars) packed tracks. Notably, two CpG islands were predicted using the UCSC genome browser and Methprimer online tool within the SPDEF genomic region (**Figures 3A, B**, green bars). Consistent with the information discovered from the above-mentioned clinical data, the first CpG island was predicted close to the PLS and the proximal enhancer (+1281 to +1619), having 400 bp and 14 CpG sites spanning +1010 to +1410 loci. The second CpG island (214bp, +6474 to +6688) was predicted within the gene body spanning exon 2 containing 18 CpG sites. Having this information, we designed primers to reveal the DNA methylation levels at single nucleotide resolution via bisulfite sequencing technique in LNCaP, PC3, DU145, and RC77/T cells. Our analysis of the resulting sequences demonstrates that SPDEF is remarkably hypomethylated in all colonies sequenced by the primer amplifying the CpG island associated with the proximal enhancer in LNCaP cells (the average percentage of methylated CpG sites in all sequenced colonies=1.4%) (**Figure 3C**, left panel). This cell line also showed a relatively lower methylation level at the distal enhancer (62.5%) than the other cell lines. Consistent with the SPDEF expression in PC3 cells, the colonies showed a relatively greater methylation level at proximal (20%) (p=0.2168) and hypermethylation at distal enhancer (91.3%, p=0.2879). Although the Mann-Whitney-U-test reported these changes as non-significant. Interestingly, most of the colonies in DU145 and RC77/T cells that do not have any expression of SPDEF showed dramatic hypermethylation at both proximal (p=0.0079 and p=0.0173, respectively) and distal (p=0.0158 and p=0.0466, respectively) enhancers (**Figure 3C**). To statistically compare the methylation status at each CpG site separately between our experimental groups (here, PC3, Du145, and RC77/T cells each versus LNCaP cells, we used Fisher’s exact test. This test showed that the DU145 and RC77/T cells are significantly hypermethylated at most CpG sites across the proximal enhancer (**Supplementary Tables S3, S4**). Also, DU145 cells are significantly hypermethylated at CpG #18, and RC77/T cells are hypermethylated at CpG #1 and CpG #18 at the distal enhancer. Our data also show significant hypermethylation of SPDEF proximal enhancer for castration-resistant LNCaP derivative cell lines, C4, C4-2, and C4-2B (**Supplemental Figure S2**). A full description of statistical data is shown in **Supplementary Materials** (**Supplementary Tables S2–S7**). In addition, the qRT-PCR analysis showed increased levels of DNMT1 mRNA in PC3, DU-145, and RC77/T cells (n=3, p<0.0001) compared to LNCaP cells (**Figure 3D**). Also, DNMT3B, the most involved enzyme in *de novo* DNA methylation, was enriched in RC77/T cells (n=3, p<0.0001). These *in vitro* data confirm the association between SPDEF levels and DNA methylation at CpG islands throughout the gene.

### 3.4 DNMT inhibition is cytotoxic to prostate cancer cells

**Figure 4:**
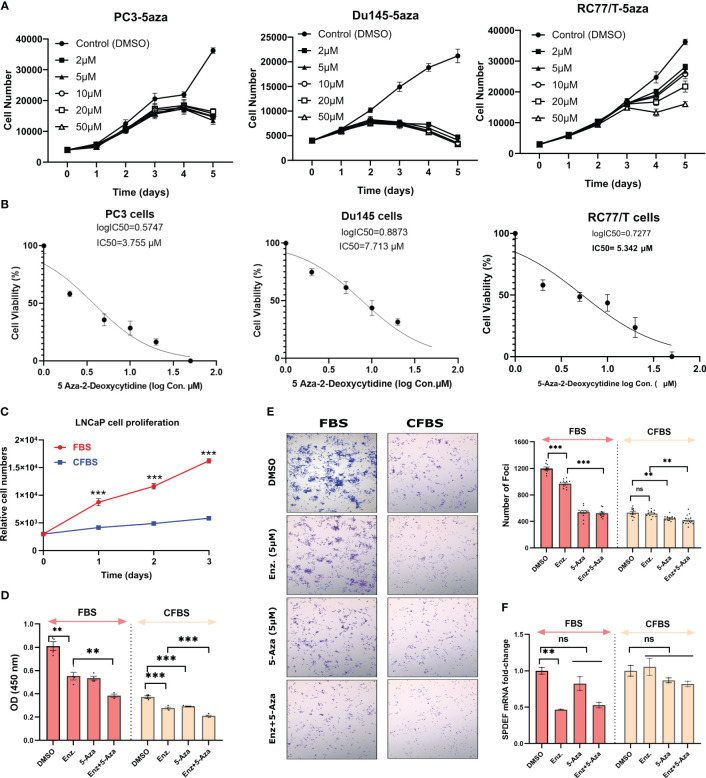
Treatment with 5Aza-dC leads to cellular toxicity in cancer cells: **(A)** 5Aza-dC dose-response in PC3, DU145, and RC77/T cells for five days. **(B)** The IC-50 curve of 5-Aza-dC on these cells. **(C)** LNCaP cell proliferation rate grown in FBS and CFBS media. **(D)** The effect of Enzalutamide (5µM), 5Aza-dC (5µM), and a combination of both agents after 72 hours of treatment on LNCaP cell growth cultured either in FBS or CFBS medium. **(E)** Crystal violet staining of foci formation assay evaluating the effect of Enzalutamide (5µM), 5Aza-dC (5µM), and a combination of both agents after 72 hours of treatment on LNCaP cells cultured either in FBS or CFBS medium (left) and Image J quantified graph (right). **(F)** SPDEF mRNA values after 72 hours of treatment with Enzalutamide (5µM), 5Aza-dC 5µM), and a combination of both agents on LNCaP cultured either in FBS or CFBS medium. ns, non-significant; p<0.01**, p<0.001***. ns, not significant.

Since we observed that aggressive PCa cell lines are highly methylated at SPDEF cis-regulatory elements, we hypothesized that blocking DNA methylation via DNA methyltransferase inhibitor, 5-Aza-2-deoxycytidine (5-Aza-dC) might be able to mimic the phenotypic changes similar to those we observed in RC77/T-SPDEF overexpression. To question this, we initially treated PC3, DU145, and RC77/T cells with different dosages of 5-Aza-dC for five days and measured the dose response in these cells. These data confirmed that 5-Aza-dC decreases cellular proliferation on all of the tested cell lines in a time and dose-dependent manner (**Figure 4A**), suggesting that the aggressive PCa lines depend on DNMTs activity for survival. The IC-50 curve of 5aza-dC on these cells is depicted in **Figure 4B**.

Next, we investigated whether treatments with 5-Aza-dC were selective and specific for PCa cells lacking SPDEF expression. We evaluated the effects of 5aza-dC in LNCaP cells, a line of PCa cells that express high levels of SPDEF. These cells also express high amounts of SPDEF protein (**Figures 1A–C**). We observed that by growing LNCaP cells on charcoal-stripped FBS (CFBS), which is deprived of androgen, the cells showed a dramatic decrease in growth rate (**Figure 4C**) measured by the CCK-8 assay. LNCaP cells are known to be remarkably dependent on the androgen receptor (AR) for growth and initially respond very well to androgen deprivation and Enzalutamide, but eventually, resistant clones develop, and these cells progress to CRPC. Since we observed that CRPC cells are sensitive to high levels of 5-Aza-dC. Therefore, we chose to test whether DNMT inhibition using 5-Aza-dC can sensitize the LNCaP cells to Enzalutamide, the first line of therapy for castration resistance PCa (CRPC). Interestingly, our foci formation assay revealed that although Enzalutamide effectively decreases foci formation in LNCaP cells grown in regular medium (p<0.0001), the effects of enzalutamide treatment in cells grown in androgen-deprived conditions were not significant (**Figure 4E**). More importantly, our data demonstrate that 5-Aza-dC either alone or in combination with Enzalutamide more efficiently suppresses the foci formation (**Figure 4E**). The foci formation results presented in average size of foci and % area are shown in **Supplementary Figure S3**. We observed that both Enzalutamide and 5-Aza-dC alone significantly inhibit cellular proliferation either in FBS or CFBS-grown LNCaP cells (**Figure 4D**). Moreover, the combination of both inhibitors offered a significantly greater reduction in cellular proliferation, especially in the CFBS medium (**Figure 4D**). Afterwards, we questioned whether growing the cells in androgen deprived condition would affect SPDEF expression levels and whether 5-Aza-dC treatment could recover its expression if suppressed. As illustrated in **Figure 4F**, our findings revealed a significant reduction in mRNA levels of SPDEF (p=0.0072) upon enzalutamide treatment in a regular medium. However, when combined with 5-aza, SPDEF levels increased to a level that was not significantly different from the vehicle-treated cells (p=0.235). On the other hand, the cells grown in CFBS did not show any further changes in SPDEF mRNA levels with either Enzalutamide, 5-aza, or a combination (**Figure 4F**, p>0.05). These data demonstrate PCa cells’ potential vulnerability to DNMT inhibition and suggest that DNMT inhibitors could be an excellent addition to current treatment regimens for prostate cancer.

### 3.5 SPDEF overexpression limits cell migration and invasion in RC77/T cells

**Figure 5:**
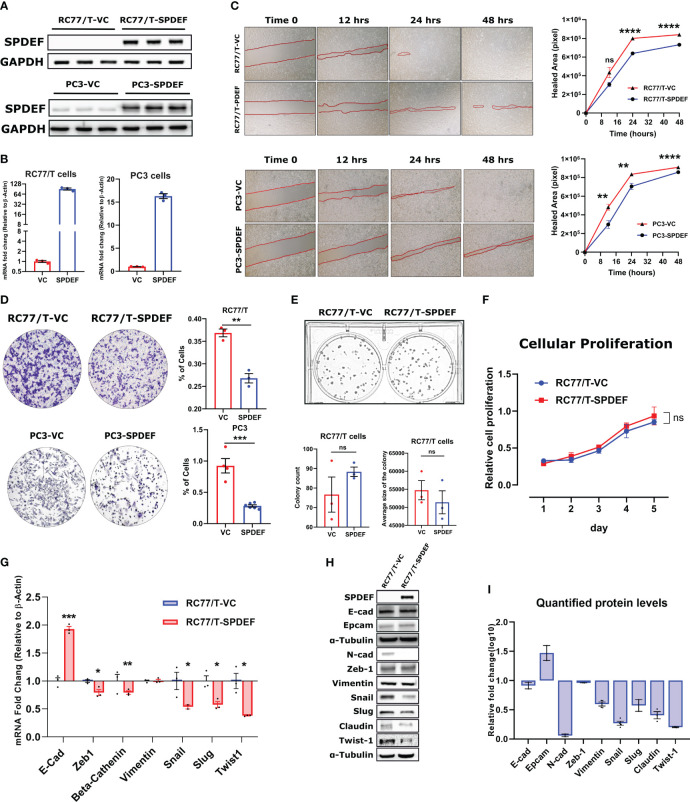
SPDEF inhibits cell migration and invasion: **(A, B)** showing the protein and mRNA levels of SPDEF in RC77/T- and PC3-VC vs. SPDEF overexpressed cells, confirming a successful stable cell line establishment. Panel **(C)** Shows the reduced migration rate in scratch (wound-healing test) in RC77/T cells (top) and PC3 (bottom) in SPDEF-overexpressed cells versus vector control cells. Panel **(D)** Shows the representative microscopic and quantified form of invasion test (Boyden chamber test) showing a reduced invasion rate in both RC77/T and PC3 overexpressing SPDEF vs vector control. Panel **(E)** Represents the colony formation test (top) and quantified representation of colony counts and the average size of the colony in each group (bottom). **(F)** Proliferation test performed on RC77/T-VC versus RC77/T-SPDEF cells. **(G)** mRNA expression of multiple EMT-related genes in RC77/T-VC versus RC77/T-SPDEF cells. **(H)** Immunoblots confirm multiple EMT-related proteins. **(I)** Shows the quantified protein levels in the immunoblot. p<0.05*, p<0.01**, p<0.001***, p<0.0001****. ns, not significant.

To functionally study the role of SPDEF in PCa progression, we generated an RC77/T line stably expressing SPDEF (RC77/T-SPDEF) and the vector control group (RC77/T-VC). We compared SPDEF expression in these cells with PC3-VC and PC3-SPDEF cells we reported earlier (27). Results (**Figures 5A, B**) show that RC77/T-SPDEF cells show robust expression of SPDEF protein and SPDEF mRNA similar to PC3-SPDEF cells compared to respective Vector control cells (**Figures 5A, B**). The PC3-VC and PC3-SPDEF lines (27) were used as comparisons for our functional study in RC77/T cells. SPDEF overexpression in RC7likey decreased cell migration in comparison to vector control (RC77/T-VC) cells at 24hrs (n=3, p=0.000003) and 48hr time points (n=3, p<0.000001 **Figure 5C**, upper panels). The results are comparable to the effects of SPDEF in the PC3 group (n=3, p<0.01 for all time points) (**Figure 5C**, lower panels). Next, we evaluated the effects of SPDEF overexpression on cell invasion in both RC77/T and PC3 groups. The cell invasion was significantly decreased in the RC77/T-SPDEF group compared to Vector control (n=3, p=0.0017, **Figure 5D**). These data were also highly consistent with the effects of SPDEF in the PC3 group (n=3, p=0.0001, **Figure 5D**). To confirm that the lower migration and invasion rates are not due to lower proliferative abilities of the SPDEF-expressing RC77/T cells, we performed colony formation and cellular proliferation assays with these cells. Interestingly, the SPDEF-expressing RC77/T cells did not show a statistically significant difference regarding proliferation (**Figure 5F**, p=0.32) rate or colony formation abilities (n=3, p=0.28, **Figure 5E**), suggesting that the effects of SPDEF on cell migration and invasion are independent of cell proliferation.

### 3.6 Overexpression of the SPDEF modulates EMT-related markers in RC77/T cells

Epithelial to mesenchymal transition (EMT) is a well-known hallmark of cancer cells undergoing metastatic/invasion stages. To test if the SPDEF can influence these markers, we measured multiple genes/proteins of the EMT panel in SPDEF expressing RC77/T cells and the Vector control group. Notably, our qRT-PCR and immunoblotting revealed that multiple EMT driver markers, including Snail, Slug, Twist1, Claudin, and N-cad, as well as genes involved in signal transduction signaling cascades such as Beta-Catenin and Zeb1, are downregulated in RC77/T-SPDEF cells (n=3, p<0.05, **Figures 5G–I**). Conversely, Vimentin did not significantly change at mRNA or protein levels (n=3, p=0.87). Interestingly, the well-known epithelial marker, E-cadherin, showed a 2-fold increase at mRNA levels (p=0.00024). Our western blot experiment did not detect any significant difference in the signal intensity for the corresponding protein. However, EpCAM, also a known epithelial marker, showed a relative increase in protein levels in RC77/T-SPDEF cells compared to vector control.

These data, taken together with the data from the clinical cohorts, suggest that loss of SPDEF during prostate cancer progression (in both Caucasian and African American) men would drive an aggressive PCa.

### 3.7 Epigenetic reprogramming of PCa cells with 5Aza-dC partially restores SPDEF expression and limits cell migration and invasion

**Figure 6:**
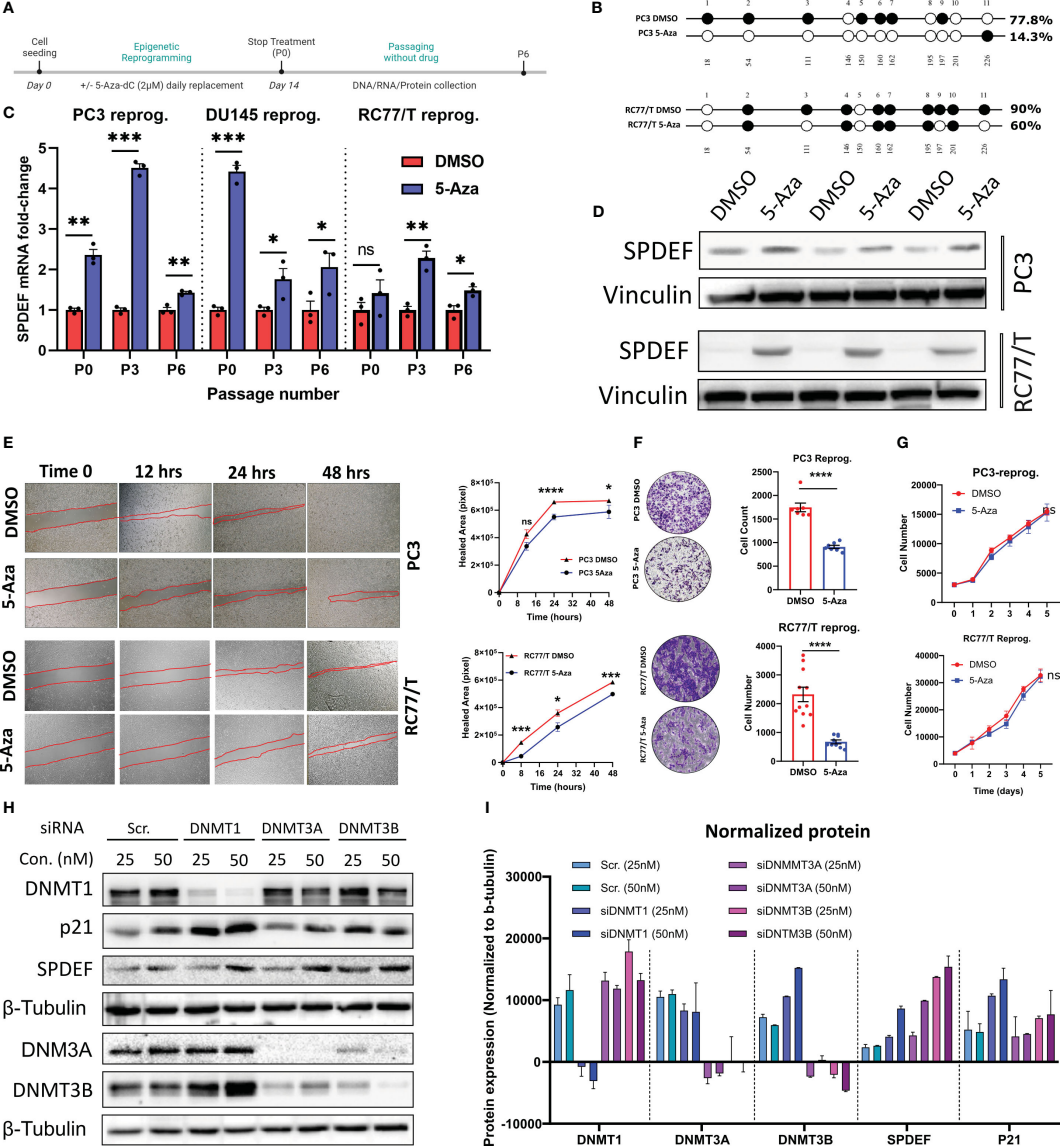
Restoration of SPDEF expression and inhibition of cell migration and invasion by Epigenetic reprogramming: **(A)** Schematic representation of the epigenetic reprogramming process. **(B)** direct sequencing of PCR results of *SPDEF* promoter indicating a successful decrease in DNA methylation after reprogramming with 5Aza-dC. **(C)** SPDEF mRNA levels on passage 0 (P0, the first day of discontinuing), passage 3 (P3), and passage 6 (Passage 6) in PC3, DU145, and RC77/T cells reprogrammed with 5Aza-dC for two weeks. Each passage number is compared with its corresponding DMSO group by t-test **(D)** Protein levels of SPDEF in reprogrammed cells vs. those treated with DMSO. **(E)** cell migration (scratch wound-healing test with quantified values of cellular migration rates (right), **(F)** Cell invasion (left) with Image J quantification of cellular invasion assay (right). **(G)** cell proliferation rate measured by MTT, **(H)** Western blot analysis of protein extracts for RC77/T cells treated with siRNA for DNMT1, DNMT3A, or DNMT3B for 72 hours. **(I)** Western blotting data quantified by Image J represented expression values normalized to beta-tubulin levels. ns, non-significant; p<0.05*, p<0.01**, p<0.001***, p<0.0001****. ns, not significant.

As shown in **Figure 4A**, RC77/T cells showed the least dramatic response to the drug compared to other cell lines PC3 and DU145 tested. These cells also express elevated levels of DNMT1 and DNMT3B levels (**Figure 3D**). Since the goal of epigenetic therapy is regulating genes through non-toxic means, we exposed PC3, DU145, and RCC7/T cells to sub-lethal doses of 5-Aza-dC (2µM) consistently for two weeks, a process known as epigenetic reprogramming (46). **Figure 6A** shows the schematic diagram for the epigenetic reprogramming experiment. Using direct sequencing of PCR products from bisulfite-treated DNA samples, we confirmed that reprogramming by 5-Aza-dC reduces DNA methylation levels on *SPDEF* promoter (**Figure 6B**). Interestingly, the cells reprogrammed by 5-Aza-dC showed significant increases in SPDEF at mRNA levels at least up to 6 passages after discontinuing the drug treatment (**Figure 6C**). Our results also demonstrate that SPDEF protein levels are upregulated in response to reprogramming by 5-Aza-dC (**Figure 6D**). We observed a non-significant difference in cellular proliferation in the cells reprogrammed by 5aza-dC versus those treated with vehicle control DMSO for the same period, suggesting that epigenetic reprogramming is non-toxic (**Figure 6G**). Our colony formation, wound healing, and invasion assays revealed that cells reprogrammed by 5-Aza-dC showed significantly reduced aggressive phenotypes compared to DMSO group. Our studies demonstrate that SPDEF expression is repressed during prostate cancer progression and CRPC development, partly by methylation on CpG islands in the SPDEF. This process could be partially reversed by DNMT inhibition.

### 3.8 SPDEF expression increases in response to siRNA-mediated DNMT targeting

In the complementary studies, we utilized small interfering RNA (siRNA) to selectively target pivotal DNA methyltransferases (DNMTs), including NDMT1, DNMT3A, and DNMT3B, with the primary objective of elucidating their roles in the modulation of SPDEF protein expression within prostate cancer cells. Our motivation was rooted in the observed phenomenon of increased SPDEF levels in response to 5aza-dC treatment, a potent general inhibitor of DNMTs. Specifically, our focus was on elucidating which class of DNMTs played a principal role in orchestrating this regulatory cascade and, critically, whether SPDEF underwent regulation through *de novo* methylation or exclusively through the maintenance of DNA methylation patterns during its loss of expression. Results of these studies (**Figures 6H, I**) demonstrate that treatment of the cells with siRNA against all DNMT classes (DMT1, DNMT3A, and DNMT3B) resulted in upregulation of SPDEF as compared to cells treated with scrambled siRNA control. This compelling evidence underscores the direct involvement of DNMTs in the regulatory machinery governing SPDEF expression in prostate cancer cells. These results also suggest that both *de novo* methylation and maintenance of DNA methylation play a role in regulating the SPDEF expression.

## 4 Discussion

This study demonstrated that CpG islands in the *SPDEF* gene are hypermethylated in CRPC cells and RC77/T cells, a line of PCa cells of African-American origin. Gene expression analyses revealed that SPDEF expression is decreased during prostate cancer progression, and there is a gradual decrease in SPDEF expression with increasing Gleason grade and a significant decrease during the transition from CRPC-Adeno to CRPC-NE. These studies agree with previous studies in prostate cancer that demonstrated reduced expression of SPDEF is correlated with tumor progression and poor patient survival (22, 25, 27, 45, 47). In contrast to these studies, two reports (48, 49) observed increased expression of SPDEF protein in prostate cancer tissues compared to normal tissues and suggested that SPDEF overexpression was associated with aggressive prostate cancer. It is interesting to point out that in the present investigation and our previous studies (27), we observed an increased expression of SPDEF mRNA in lower Gleason grade (3 + 3 and 3 + 4) compared to adjacent normal tissues. However, SPDEF levels in high Gleason-grade tumors (4 + 3 and higher) are significantly lower compared to low-grade tumors. Additional studies are required to resolve these controversies. Notwithstanding these discrepancies, given that SPDEF is a luminal-epithelial specific transcription factor, loss of SPDEF expression during prostate cancer progression suggests dedifferentiation away from luminal-epithelial phenotype and cellular plasticity. This notion is further supported by the results of SPDEF expression in RC77/T cells (**Figure 5**) and previous studies that demonstrate that the expression of SPDEF in several cancer cell lines, including prostate cancer (50, 51), decreased cell migration and invasion in part by limiting expression of EMT markers and promoting luminal differentiation (27), consistent with tumor metastasis suppressor role for SPDEF in prostate cancer as proposed previously (23, 24, 27).

Spatial, temporal regulation of gene expression depends not only on the presence or absence of transcription factors that turn the gene on or off but also requires accessibility of the transcription factor binding sites on the DNA. We observed that CpG-islands in the *SPDEF* gene are hypermethylated in prostate cancer cells that do not express SPDEF (**Figure 3**). Like prostate cancer cell lines, we observed that methylation at CpG islands of the *SPDEF* gene in prostate cancer patient tissues is inversely correlated with SPDEF expression and the extent of methylation at the CpG sites (**Figure 2**). These results are consistent with the existing literature that cytosine methylation on CpG dinucleotides serves as a causal mechanism for transcriptional repression at cis-regulatory elements, and a correlation between DNA methylation and gene expression has been established (52). DNA methylation also leads to gene-silencing by limiting access of transcription factors and RNA polymerases to target binding sites (31, 53) or by recruitment of methyl-binding domain proteins, which facilitate the assembly of chromatin condensation into transcriptionally repressive conformations (32, 33). It is important to note that alterations in DNA methylation have been documented in PCa (29, 30) and have been shown to suppress several genes, including E-Cadherin and Androgen Receptor (54, 55).

DNA methylation is catalyzed by DNA methyltransferases (DNMTs), including DNMT1, DNMT3a, and DNMT3b (56). DNMT1 is required to maintain the specific methylation pattern during replication, while DNMT3A and DNMT3B are responsible for establishing DNA methylation patterns during embryogenesis (57). Although they are highly expressed in early mammalian embryos, DNMT3A and DNMT3B decrease in expression throughout cell differentiation (58). Our results show that DNMT1, DNMT3A, and DNMT3B are expressed, albeit to a different extent, in all the PCa cell lines tested. Moreover, we demonstrate that all three DNMT levels correlate with SPDEF at the mRNA level in the clinical data sets in prostate cancer tissues. There is a direct relationship between DNMT expression and methylation on the CpG islands in the *SPDEF* gene and an inverse relation between DNMT expression and SPDEF expression. However, given that SPDEF is not the only gene regulated by DNA methylation, it is vital to understand how the DNMTs are recruited to the *SPDEF* gene. Moreover, given that DNMT3A and DNMT3B are expressed explicitly during embryogenesis (57), our data point to the reactivation of embryogenic programs in PCa.

Previous studies have demonstrated that treatment of cancer cells with 5Aza-dC (a pan DNMT inhibitor) (10µM) resulted in the restoration of expression of genes with hypermethylated CpG islands in the promoter region. Our results show that 5Aza-dC elicited cytotoxicity in CRPC cells in a dose-dependent fashion with an IC50 of ~5µM (**Figure 4**). These data suggested that CRPC cells could be preferentially dependent on DNMT activities for survival. However, castrate-sensitive LNCaP cells were equally susceptible to 5Aza-dC. We discovered that 5Aza-dC (5µM) was more effective in reducing the growth and proliferation of LNCaP Cells than AR modulating agent Enzalutamide.

Moreover, 5Aza-dC treatment also sensitized LNCaP cells to Enzalutamide. These results demonstrate a critical requirement for DNMTs in therapeutic resistance to AR-targeted therapies. Because resistance to AR-targeted therapies is driven, in part, by cellular plasticity, we posit that DNMTs could serve as potentially actionable targets to limit therapy-driven cellular plasticity in prostate cancer. The mechanisms that drive these effects require further investigation.

Reversing epigenetic changes offers a unique chance for cancer cell reprogramming, which is essential for developing new therapeutics. We exposed PC3, DU145, and RCC7/T cells to sub-lethal doses of 5aza-dC (2µM) for two weeks. This experiment aimed to alter the phenotype of these cells to less aggressive ones. The technique used is known as “epigenetic reprogramming” (46). We measured SPDEF expression levels, cell migration, and invasion following treatment with non-cytotoxic dosages of the demethylating chemical 5aza-dC. We observed that long-term exposure of CRPC cells to sub-lethal doses of 5aza-dC (2µM) resulted in partial restoration of SPDEF expression (**Figure 6**). SPDEF expression remained significantly elevated for at least six passages after discontinuing the drug. These treatments also decreased cell migration and invasion in RC77/T cells, similar to those observed with SPDEF overexpression (**Figure 5**). Our findings demonstrate that 5-Aza-dC-mediated reprogramming and SPDEF expression enhancement can be replicated in aggressive prostate cancer cell lines by depleting DNMT1. DNA demethylating agents represent a promising alternative for treating solid tumors, as seen in our study, where 5-Aza-dC reduced invasive phenotypes of PCa cells while increasing response to enzalutamide (**Figure 4**). These data suggested that CRPC cells could be preferentially dependent on DNMT activities for survival. Previous research has shown the reactivation of specific genes by hypomethylation medications as key to therapeutic benefits in various cancers, including melanoma (59), glioma (60), and non-small cell lung cancer (61). However, the ideal dosage and potential adverse effects of demethylating agents still require further investigation in PCa. However, it will be crucial to establish the selectivity of cancer cell reprogramming and understand why cancer-imprinted genes are prone to reactivation by demethylation treatment. Our investigation into the impact of siRNA-mediated targeting of DNMTs (NDMT1, DNMT3A, and DNMT3B) on SPDEF expression in prostate cancer cells has yielded valuable insights. Motivated by the observed increase in SPDEF levels upon treatment with 5aza-dC, a broad DNMT inhibitor, our study aimed to discern the specific roles of DNMT classes in SPDEF regulation and whether this modulation involved *de novo* methylation or maintenance of DNA methylation patterns. Our results unequivocally confirm the direct involvement of DNMTs in governing SPDEF expression, as evidenced by a significant upregulation in SPDEF protein levels following siRNA treatment against all DNMT classes.

In summary, our studies demonstrate that SPDEF expression is decreased during prostate cancer progression and CRPC development, partly by methylation on CpG islands in the *SPDEF* gene. This process could be partially reversed by treatment with DNMT inhibition. We speculate that 5aza-dC and other DNMT inhibitors might serve as excellent epigenetic modifiers and adjuvants to our current therapeutic regimens to limit cellular plasticity in prostate cancer.

## Data availability statement

The original contributions presented in the study are included in the article/**Supplementary Material**. Further inquiries can be directed to the corresponding author.

## Ethics statement

The studies involving humans were approved by IBC, LSUHSC, New Orleans, LA-70112. The studies were conducted in accordance with the local legislation and institutional requirements. The human samples used in this study were acquired from Cell lines commercially available. Written informed consent for participation was not required from the participants or the participants’ legal guardians/next of kin in accordance with the national legislation and institutional requirements.

## Author contributions

HK: Conceptualization, project administration. MV, HK, and LC: writing-original draft preparation. HK, SK, LC, AO, and JS: Editing. MV, LC, SK, HK, and JS: Investigation and writing-Manuscript. All authors contributed to the article and approved the submitted version.
