# Supplementary material for: Regulation of SPDEF expression by DNA methylation in advanced prostate cancer
Source: Front Endocrinol (Lausanne). 2023 Oct 11;14:1156120. doi: 10.3389/fendo.2023.1156120 (PMC10600024; doi:10.3389/fendo.2023.1156120)
Supplement: Supplementary file 1 [file DataSheet_1.pdf]

Supplemental Materials

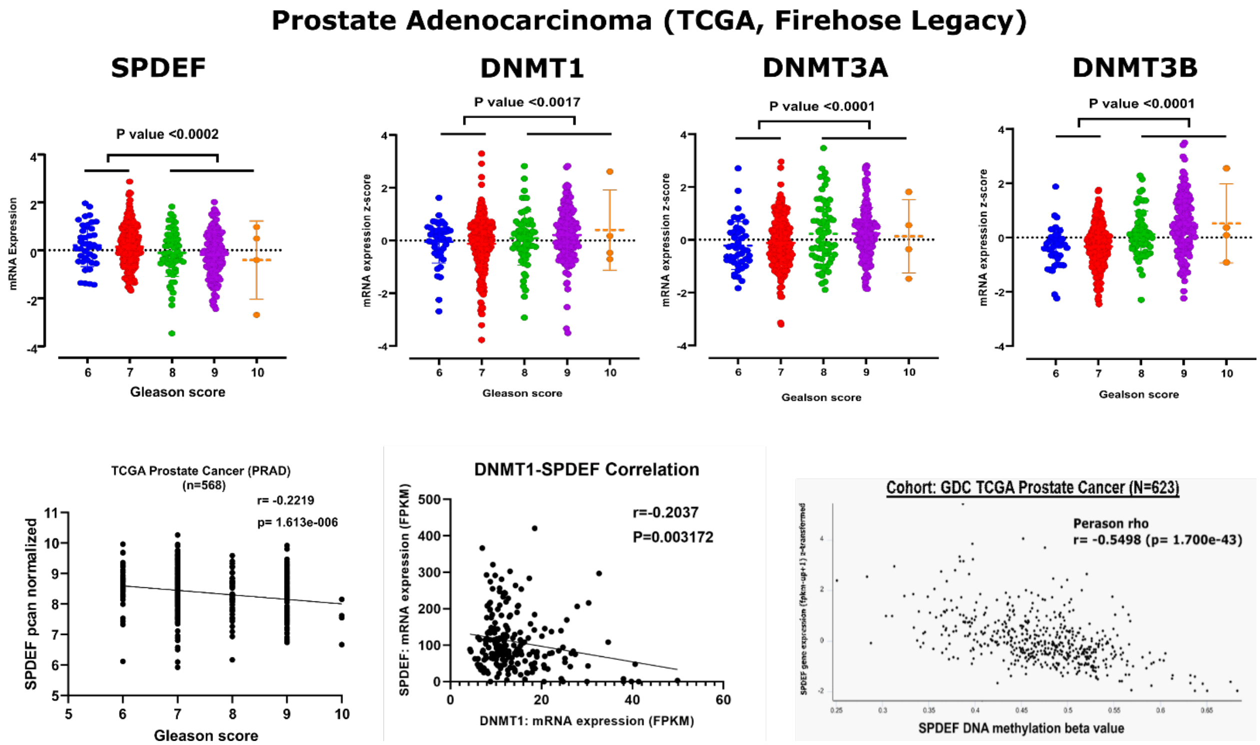

Figure S1. Analysis of multiple other datasets from various clinical cohorts (mentioned on top of each panel set) showed a significantly lower mRNA values for SPDEF and significantly greater values for DNA methyltransferase (DNMTs) in High-Gleason score patients versus lower grades (Top panel). Correlation analysis performed on TCGA prostate cancer (PRAD) cohort demonstrated a significant negative correlation between SPDEF levels and Gleason score (lower left), and a negative correlation between DNMT1 and SPDEF level (lower middle). Analysis of GDC TCGA Prostate Cancer cohort also showed a negative correlation between SPDEF mRNA levels and the overall methylation beta value across the SPDEF gene.

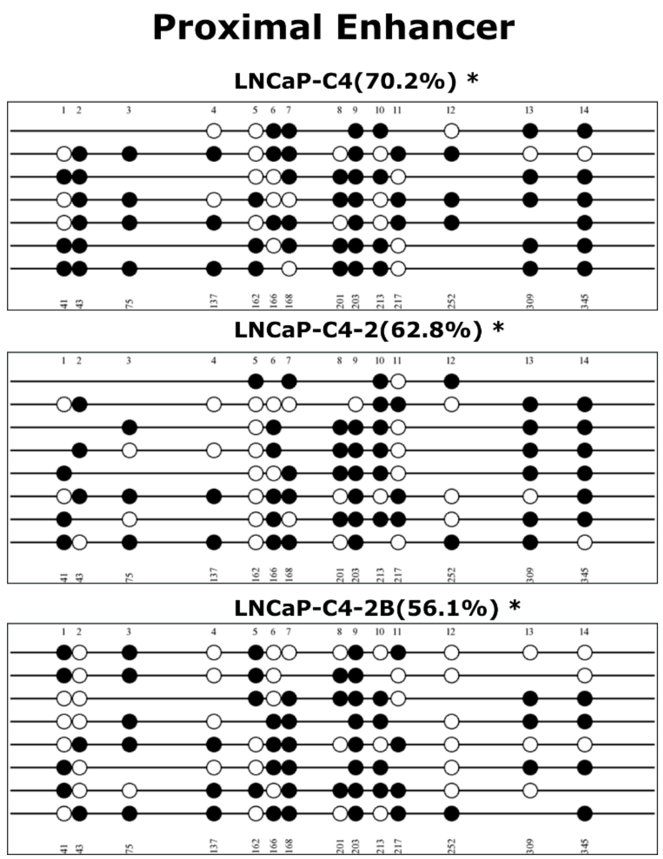

Figure S2. Bisulfite sequencing (BSP) experiment on castration-resistant LNCaP-derived cells (C4, C4-2, and C4-2B) showed a significant hypermethylation on SPDEF proximal enhancer region in the androgen-independent cells versus LNCaP cells

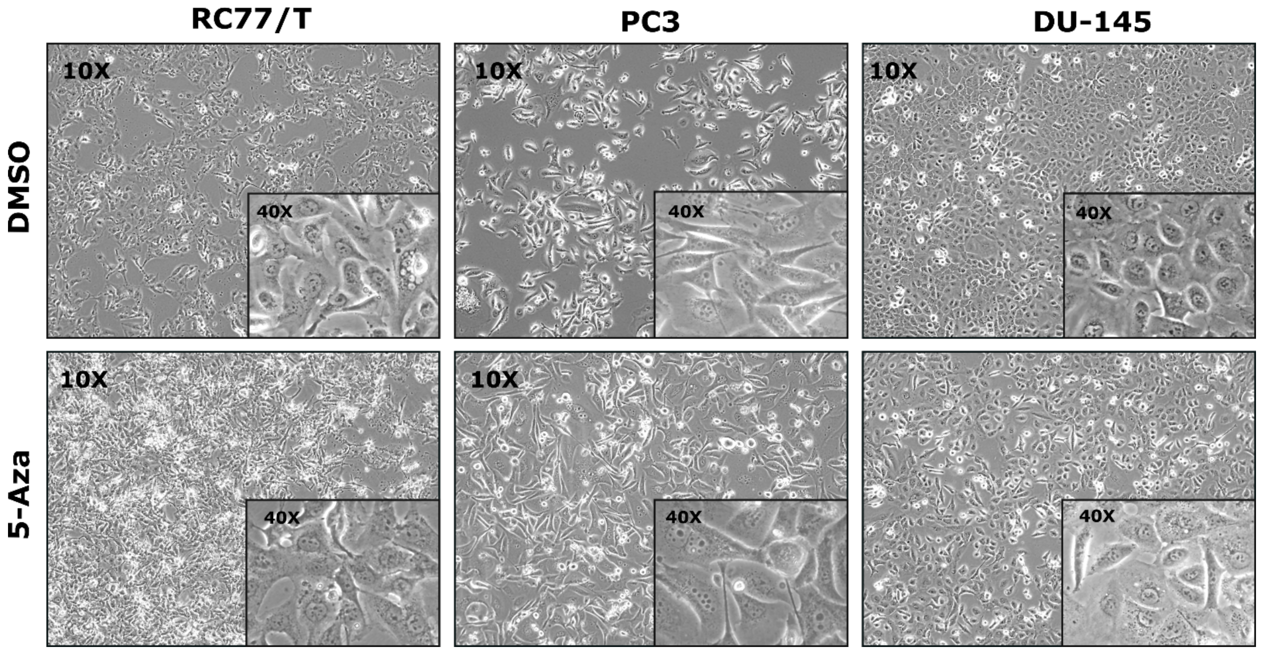

Figure S3. Morphological view of the RC77/T, PC3, and DU145 cells reprogrammed with 5-aza or DMSO as control. Each panel depicts the microscopic views of 10X and 40X magnification.

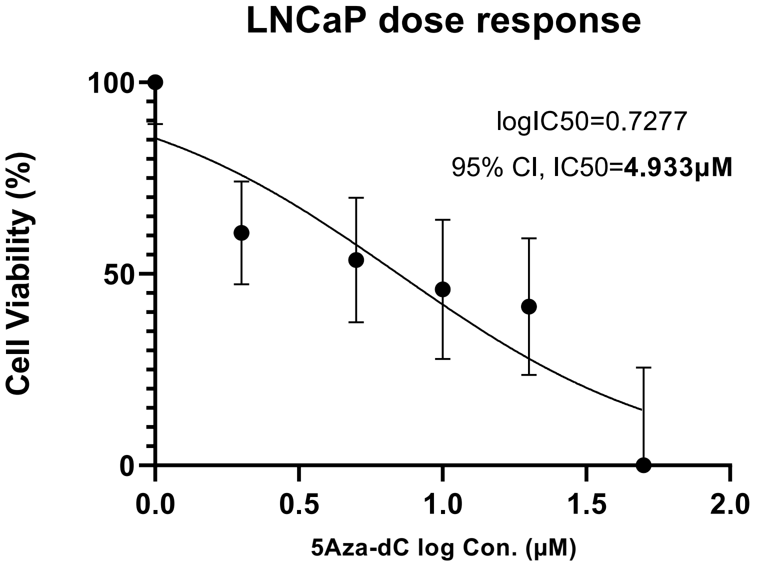

Figure S4. IC50 of 95% CI from 72 hours' treatment of LNCaP cells by 5Aza-dC.

Table S1. The primer sequences used in BSP and qRT-PCR techniques.

Gene name (Accession Number)	Forward sequence (5' to 3')	Reverse sequence (5' to 3')
**SPDEF BSP proximal enhancer**	TTTGGTTTTGGTATATAAGGAAAG	AATCTTAACCTACCCCTCCC
**SPDEF BSP distal enhancer**	GGGTATAGTATGTTAAAGTAGGAGAGG	AATCAAACCTAAATCCTCTTTCA
**TWIST1 (NM_000474)**	GCCAGGTACATCGACTTCCTCT	TCCATCCTCCAGACCGAGAAGG
**Vimentin (VIM) (NM_003380)**	AGGCAAAGCAGGAGTCCACTGA	ATCTGGCGTTCCAGGGACTCAT
**E Cadherin (CDH1) (NM_004360)**	GCCTCCTGAAAAGAGAGTGGAAG	TGGCAGTGTCTCTCCAAATCCG
**N Cadherin (CDH2)(NM_001792)**	CCTCCAGAGTTTACTGCCATGAC	GTAGGATCTCCGCCACTGATTC
**SNAIL (SNAI1) (NM_005985)**	TGCCCTCAAGATGCACATCCGA	GGGACAGGAGAAGGGCTTCTC
**SLUG (SNAI2) (NM_003068)**	ATCTGCGGCAAGGCGTTTTCCA	GAGCCCTCAGATTTGACCTGTC
**AREB6 (ZEB1) (NM_030751)**	GGCATACACCTACTCAACTACGG	TGGGCGGTGTAGAATCAGAGTC
**SPDEF (NM_012391)**	CGAAGTGCTCAAGGACATCGAG	CGGTATTGGTGCTCTGTCCACA
**Beta Catenin (NM_001098209)**	CACAAGCAGAGTGCTGAAGGTG	GATTCCTGAGAGTCCAAAGACAG
**Beta Actin (ACTB) (NM_001101)**	CACCATTGGCAATGAGCGGTTC	AGGTCTTTGCGGATGTCCACGT

Table S2. Methylation status of each CpG site at proximal Enhancer (LNCaP vs PC3)

CpG position	41	43	75	137	162	166	168	201	203	213	217	252	309	345	Total
Number of methylated CpGs (LNCaP)	0	0	0	0	0	0	1	0	0	0	0	0	0	0	1
Number of CpGs (LNCaP)	5	5	5	5	5	5	5	5	5	5	5	5	5	4	69
ratio of methylation (%) (LNCaP)	0	0	0	0	0	0	20	0	0	0	0	0	0	0	1.44
Number of methylated CpGs (PC3)	2	2	1	1	1	2	3	3	3	4	1	0	3	3	29
Number of CpGs (PC3)	8	8	7	7	6	7	7	6	7	7	6	7	8	8	99
ratio of methylation (%) (PC3)	25	25	14.3	14.3	16.7	28.6	42.9	50	42.9	57.1	16.7	0	37.5	37.5	29.29
Number of methylated CpGs (total)	2	2	1	1	1	2	4	3	3	4	1	0	3	3	30
Number of CpGs (total)	13	13	12	12	11	12	12	11	12	12	11	12	13	12	168
ratio of methylation (%) (total)	15.4	15.4	8.3	8.3	9.1	16.7	33.3	27.3	25	33.3	9.1	0	23.1	25	17.85
P-value of Fisher's exact test	0.487	0.487	1	1	1	0.469	0.575	0.181	0.204	0.080	1	1	0.230	0.490	2.57E-06
P-value of Mann-	0.216														

Whitney U-test

Table S3. Methylation status of each CpG site at proximal Enhancer (LNCaP vs DU145)

CpG position	41	43	75	137	162	166	168	201	203	213	217	252	309	345	Total
Number of methylated CpGs (LNCaP)	0	0	0	0	0	0	1	0	0	0	0	0	0	0	1
Number of CpGs (LNCaP)	5	5	5	5	5	5	5	5	5	5	5	5	5	4	69
ratio of methylation (%) (LNCaP)	0	0	0	0	0	0	20	0	0	0	0	0	0	0	1.449
Number of methylated CpGs (DU145)	5	2	4	5	4	5	5	5	2	5	2	0	5	2	51
Number of CpGs (DU145)	5	4	5	5	5	5	5	5	5	5	5	5	5	5	69
ratio of methylation (%) (DU145)	100	50	80	100	80	100	100	100	40	100	40	0	100	40	73.91
Number of methylated CpGs (total)	5	2	4	5	4	5	6	5	2	5	2	0	5	2	52
Number of CpGs (total)	10	9	10	10	10	10	10	10	10	10	10	10	10	9	138
ratio of methylation (%) (total)	50	22.2	40	50	40	50	60	50	20	50	20	0	50	22.2	37.681
P-value of Fisher's exact test	0.007	0.166	0.047	0.007	0.047	0.007	0.047	0.007	0.444	0.007	0.444	1	0.007	0.444	6.75E-21
P-value of Mann-Whitney U-test	0.007														

Table S4. Methylation status of each CpG site at proximal Enhancer (LNCaP vs RC77/T)

CpG position	41	43	75	137	162	166	168	201	203	213	217	252	309	345	Total
Number of methylated CpGs (LNCaP)	1	1	1	1	1	0	1	0	1	1	0	0	0	0	8
Number of CpGs (LNCaP)	6	6	6	6	6	5	6	5	6	6	6	5	6	5	80
ratio of methylation (%) (LNCaP)	16.7	16.7	16.7	16.7	16.7	0	16.7	0	16.7	16.7	0	0	0	0	10
Number of methylated CpGs (RC77/T)	2	7	2	5	5	4	5	6	6	6	6	4	6	5	69
Number of CpGs (RC77/T)	7	7	6	6	7	6	7	6	7	7	7	6	7	7	93
ratio of methylation (%) (RC77/T)	28.6	100	33.3	83.3	71.4	66.7	71.4	100	85.7	85.7	85.7	66.7	85.7	71.4	74.19
Number of methylated CpGs (total)	3	8	3	6	6	4	6	6	7	7	6	4	6	5	77
Number of CpGs (total)	13	13	12	12	13	11	13	11	13	13	13	11	13	12	173
ratio of methylation (%) (total)	23.1	61.5	25	50	46.2	36.4	46.2	54.5	53.8	53.8	46.2	36.4	46.2	41.7	44.50
P-value of Fisher's exact test	1	0.004	1	0.080	0.102	0.060	0.102	0.002	0.029	0.029	0.004	0.060	0.004	0.027	1.56E-18
P-value of Mann-Whitney U-test	0.017														

Table S5. Methylation status of each CpG site at distal Enhancer (LNCaP vs PC3)

CpG position	8	23	27	32	55	62	78	84	103	105	111	116	146	159	167	179	197	209	Total
Number of methylated CpGs (LNCaP)	2	3	3	5	5	3	3	3	3	3	3	3	3	3	3	3	3	1	55
Number of CpGs (LNCaP)	5	3	5	5	5	5	5	5	5	5	5	5	5	5	5	5	5	5	88
ratio of methylation (%) (LNCaP)	40	100	60	100	100	60	60	60	60	60	60	60	60	60	60	60	60	20	62.5
Number of methylated CpGs (PC3)	4	7	7	7	6	7	6	7	7	6	6	6	7	7	7	7	6	5	115
Number of CpGs (PC3)	7	7	7	7	7	7	7	7	7	7	7	7	7	7	7	7	7	7	126
ratio of methylation (%) (PC3)	57.1	100	100	100	85.7	100	85.7	100	100	85.7	85.7	85.7	100	100	100	100	85.7	71.4	91.26
Number of methylated CpGs (total)	6	10	10	12	11	10	9	10	10	9	9	9	10	10	10	10	9	6	170
Number of CpGs (total)	12	10	12	12	12	12	12	12	12	12	12	12	12	12	12	12	12	12	214
ratio of methylation (%) (total)	50	100	83.3	100	91.7	83.3	75	83.3	83.3	75	75	75	83.3	83.3	83.3	83.3	75	50	79.43
P-value of Fisher's exact test	1	1	0.15	1	1	0.15	0.52	0.15	0.15		0.52	0.52	0.15	0.15	0.15	0.15	0.52	0.24	3.86E-07
P-value of Mann-Whitney U-test	0.28																		

Table S6. Methylation status of each CpG site at distal Enhancer (LNCaP vs DU145)

CpG position	8	23	27	32	55	62	78	84	103	105	111	116	146	159	167	179	197	209	Total
Number of methylated CpGs (LNCaP)	2	3	3	5	5	3	3	3	3	3	3	3	3	3	3	3	3	1	55
Number of CpGs (LNCaP)	5	3	5	5	5	5	5	5	5	5	5	5	5	5	5	5	5	5	88
ratio of methylation (%) (LNCaP)	40	100	60	100	100	60	60	60	60	60	60	60	60	60	60	60	60	20	62.5
Number of methylated CpGs (DU145)	5	5	5	5	5	5	5	5	5	5	5	5	5	5	5	5	4	5	89
Number of CpGs (DU145)	5	5	5	5	5	5	5	5	5	5	5	5	5	5	5	5	5	5	90
ratio of methylation (%) (DU145)	100	100	100	100	100	100	100	100	100	100	100	100	100	100	100	100	80	100	98.88
Number of methylated CpGs (total)	7	8	8	10	10	8	8	8	8	8	8	8	8	8	8	8	7	6	144
Number of CpGs (total)	10	8	10	10	10	10	10	10	10	10	10	10	10	10	10	10	10	10	178
ratio of methylation (%) (total)	70	100	80	100	100	80	80	80	80	80	80	80	80	80	80	80	70	60	80.89
P-value of Fisher's exact test	0.16	1	0.44	1	1	0.44	0.44	0.44	0.44	0.44	0.44	0.44	0.44	0.44	0.44	0.44	1	0.04	4.24E-11
P-value of Mann-Whitney U-test	0.015873																		

Table S7. Methylation status of each CpG site at distal Enhancer (LNCaP vs RC77/T)

CpG position	8	23	27	32	55	62	78	84	103	105	111	116	146	159	167	179	197	209	Total
Number of methylated CpGs (LNCaP)	2	3	3	5	5	3	3	3	3	3	3	3	3	3	3	3	3	1	55
Number of CpGs (LNCaP)	5	3	5	5	5	5	5	5	5	5	5	5	5	5	5	5	5	5	88
ratio of methylation (%) (LNCaP)	40	100	60	100	100	60	60	60	60	60	60	60	60	60	60	60	60	20	62.5
Number of methylated CpGs (RC77/T)	8	8	8	8	8	6	8	8	8	8	5	8	8	7	7	7	8	8	136
Number of CpGs (RC77/T)	8	8	8	8	8	8	8	8	8	8	8	8	8	7	8	8	8	8	143
ratio of methylation (%) (RC77/T)	100	100	100	100	100	75	100	100	100	100	62.5	100	100	100	87.5	87.5	100	100	95.10
Number of methylated CpGs (total)	10	11	11	13	13	9	11	11	11	11	8	11	11	10	10	10	11	9	191
Number of CpGs (total)	13	11	13	13	13	13	13	13	13	13	13	13	13	12	13	13	13	13	231
ratio of methylation (%) (total)	76.9	100	84.6	100	100	69.2	84.6	84.6	84.6	84.6	61.5	84.6	84.6	83.3	76.9	76.9	84.6	69.2	82.68
P-value of Fisher's exact test	0.03	1	0.12	1	1	1	0.12	0.12	0.12	0.12	1	0.12	0.12	0.15	0.51	0.51	0.12	0.006	3.22E-10
P-value of Mann-Whitney U-test	0.046																		
